# Method of relieving hypertoxic alcohol abuse states in alcohol dependence

**DOI:** 10.1192/j.eurpsy.2021.1501

**Published:** 2021-08-13

**Authors:** I. Sosin, Y. Babenko, O. Sergienko, O. Honcharova, G. Mysko, I. Lisova

**Affiliations:** 1 Narcology Department, Kharkiv, Kharkiv Medical Academy of Postgraduate Education, Kharkiv, Ukraine; 2 Narcology Department, Kharkiv Medical Academy of Postgraduate Education, Kharkiv, Ukraine

**Keywords:** Alcohol addiction, Intoxication, Treatment

## Abstract

**Introduction:**

Currently, alcohol dependence is characterized by immediate onset of dipsomania states (code F10.26, ICD-10) interpreted in clinical addictology as reliable diagnostic signs of morbid alcohol dependence. These are classified clinically by rate, severity, therapeutically resistant post alcohol comorbidities (alcohol-induced polyneuropathy, hepatic dysfunctions, etc.), and by the presence of “lucid spaces”, when patients, depleted physically and mentally by hypertoxic alcohol abuse states, periodically (after binge drinking) intake no alcohol.

**Objectives:**

Effectiveness improvement and reducing time of treatment for hypertoxic alcohol abuse states by reasonable pathogenetic use of highly effective drugs, wide polymodality and synergistic pharmacological range, with few side effects, potential for inclusion to the conventional standard treatment patterns according to thiamine concepts.

**Methods:**

Valid clinical-diagnostic, laboratory, biochemical, electrophysiological, psychological (scaling, testing), statistical methods for identification of alcohol dependence complicated by hypertoxic alcohol abuse states.

**Results:**

A new method of alleviating the hypertoxic intoxication in alcohol dependence has been developed on representative clinical material, which involves conventional pharmacological and drug-free symptomatic remedies and methods. Along with psychotherapeutic potentiation, a therapeutically targeted pharmacological complex was prescribed: intramuscular Vitaxon № 10 per course; Sibazon 0.5% solution, 2 ml intramuscular, 3-5 injections per course; oral Phenazepam, one tablet (0.001g) twice a day for 10-14 days; Cocarnit one ampoule daily intramuscular injection, for a course of 3-10 injections.

**Conclusions:**

The effectiveness of the proposed pharmacological complex has been proven by the statistical reliability method and illustrated by clinical examples of patient-specific research.

